# An integration of deep learning with feature embedding for protein–protein interaction prediction

**DOI:** 10.7717/peerj.7126

**Published:** 2019-06-17

**Authors:** Yu Yao, Xiuquan Du, Yanyu Diao, Huaixu Zhu

**Affiliations:** The School of Computer Science and Technology, Anhui University, Hefei, Anhui, China

**Keywords:** Deep learning, protein–protein interaction, Feature embedding, Machine learning

## Abstract

Protein–protein interactions are closely relevant to protein function and drug discovery. Hence, accurately identifying protein–protein interactions will help us to understand the underlying molecular mechanisms and significantly facilitate the drug discovery. However, the majority of existing computational methods for protein–protein interactions prediction are focused on the feature extraction and combination of features and there have been limited gains from the state-of-the-art models. In this work, a new residue representation method named Res2vec is designed for protein sequence representation. Residue representations obtained by Res2vec describe more precisely residue-residue interactions from raw sequence and supply more effective inputs for the downstream deep learning model. Combining effective feature embedding with powerful deep learning techniques, our method provides a general computational pipeline to infer protein–protein interactions, even when protein structure knowledge is entirely unknown. The proposed method DeepFE-PPI is evaluated on the S. Cerevisiae and human datasets. The experimental results show that DeepFE-PPI achieves 94.78% (accuracy), 92.99% (recall), 96.45% (precision), 89.62% (Matthew’s correlation coefficient, MCC) and 98.71% (accuracy), 98.54% (recall), 98.77% (precision), 97.43% (MCC), respectively. In addition, we also evaluate the performance of DeepFE-PPI on five independent species datasets and all the results are superior to the existing methods. The comparisons show that DeepFE-PPI is capable of predicting protein–protein interactions by a novel residue representation method and a deep learning classification framework in an acceptable level of accuracy. The codes along with instructions to reproduce this work are available from https://github.com/xal2019/DeepFE-PPI.

## Introduction

Most biological processes within a cell are induced by a variety of interactions among the proteins and protein–protein interactions can form the basis for understanding protein functions, communications and regulations. Due to their importance in the cell, many experiential and computational methods have been developed to identify various interactions and determine the aftermath of the interactions (Browne et al., 2010).

High-throughput techniques, such as yeast-2-hybrid (Y2H) assay, Co-Immunoprecipitation (co-IP), mass spectrometric (MS) protein complex identification, affinity purification (AP) etc., are first utilized to judge protein–protein interactions. Unfortunately, experimental approaches hold many inherent disadvantages and constraints. One of the limitations is the lack of coverage and quality of the global protein–protein interaction networks (Schoenrock et al., 2014). In addition, each method is inherently subject to different type of noise and they are mostly slow, time-consuming, and expensive. The existing shortcomings promote the emergence of a number of efficient computational methods to infer most potential protein interactions. One of the most prominent computational methods utilizes protein’s structural information. The idea behind the structure-based methods is that two proteins will interact if they have a similar structure. Lu, Lu & Skolnick (2002) rethreaded on partners for those proteins whose template structures were part of a known complex and 1,138 interactions among 2,865 protein–protein interactions in yeast had been confirmed in the Database of Interacting Proteins (DIP) (Xenarios et al., 2002). Hue et al. (2010) used statistical structure information and built a hypothesis using Support Vector Machines (SVM) to decide whether a pair of proteins interact or not. Recently, Hosur et al. (2012) introduced a structure-based algorithm by computing a single confidence score to infer thousands of binary protein interactions. A method combined three-dimensional structural information with other functional clues called PrePPI was proposed by Zhang et al. (2012) to detect protein–protein interactions and it holds a superior accuracy and coverage. PrePPI is one of the typical methods based on template structure. There also exists template free-based protein–protein interaction prediction methods. Wass et al. (2011) designed a docking algorithm to predict protein–protein interactions and presented a standard docking program to distinguish true interactions from a background of 922 non-redundant potential interactions. MEGADOCK proposed by Ohue et al. (2014) is also a template free-based method. It was capable of exhaustive protein–protein interaction screening in less calculation time and an acceptable level of accuracy and obtained an F-measure value of 0.231 when it was applied to predict 120 relevant interacting pairs from 14,400 combinations of proteins. It could also be used to search and analyze protein–protein interactions when taking into account three-dimensional.

Kundrotas et al. (2012) claimed that docking templates can be found for complexes to represent almost all the known protein–protein interactions. However, it is undeniable that the number of protein sequences is much larger than the limited number of structures. The importance of prediction based on sequence information does not change. Thanks to the rapid growth of protein sequence data, a number of machine learning methods solely on the basis of primary amino acid sequence have also been proposed to infer the interactions between proteins (Rhodes et al., 2005; Shen et al., 2007; Burger & Van Nimwegen, 2008; Xia, Han & Huang, 2010; Hosur et al., 2011; You et al., 2013; Valente et al., 2013; Martin, Roe & Faulon, 2005). For sequence-based methods, the first challenge is to find a suitable way to represent a protein sequence. The existing methods typically extract numerical features based on hydrophobicity, normalized Van der Waals volume, polarity, polarizability, charge, surface tension and solvent accessibility and so on Asgari & Mofrad (2015). For example, Shen et al. (2007) developed a conjoint triad feature extraction method and the SVM classifier reached a higher prediction 83.5% accuracy on 400 test protein pairs when trained on 32,486 human protein pairs. Xia, Han & Huang (2010) combined rotation forest and autocorrelation descriptor for protein–protein interactions prediction. When tested on S.cerevisiae datasets and H.pylori (2,916 protein pairs including 1,458 interacting pair and 1,458 non-interacting pairs), the model obtained 93.50% accuracy, 96.30% precision, 90.50% sensitivity and 88.36% accuracy, 89.19% precision, 88.20% sensitivity, respectively. You et al. (2013) used hierarchical principal component analysis-ensemble extreme learning machine to predict protein–protein interactions only using the protein sequences information. The proposed model achieved 87.00% accuracy with 86.15% sensitivity at the precision of 87.59% on a high-confidence S.cerevisiae protein–protein interaction dataset, which has 5,594 positive protein pairs and 5,594 negative protein pairs.

Although machine learning methods can use training data to build models and make predictions based on the best fit model, there still have some inherent drawbacks in raw data processing. One is that the majority of computational methods is highly dependent on hand-designed features. Another limitation is that these carefully chosen features are derived primarily from single attribute (e.g., protein structure information, protein domains, or gene neighborhoods information), rather than directly from raw data. Considering the huge amount of validated protein–protein interaction sequences produced by high-throughput technique and urgent needs to change from hand-designed to data-driven features, representation learning which can discover effective feature, underlying patterns and inherent mappings is adopted for data representation and deep learning is used to accomplish the prediction task.

In the machine learning community, representation learning and deep learning are now two popular techniques for feature generation, noise elimination and accurate prediction. Representation learning aims to automatically learn data representations from raw data, these representations can be exploited by downstream machine learning models to improve the task performance (Bengio, Courville & Vincent, 2012). Given a set of neurons and inner special connectivity patterns, deep learning can learn the features of inputs and has become one of the trendiest methods in computational biology.

Word2vec is a successful word embedding technique in natural language processing (Mikolov et al., 2013a; Mikolov et al., 2013b). The tool can discover semantic relations between words in the document. In this work, a residue representation method, named Res2vec, is reported for protein feature generation. Res2vec can embed a residue into a low-dimensional vector. Given a protein–protein pair, each sequence is expressed by Res2vec and then the represented proteins are sent into the successive machine learning framework to compute their interaction probability. We have tested our method on several benchmark datasets. The comparisons with several methods have demonstrated the superior performance of our approach for predicting new protein–protein interactions, especially when the structure knowledge of proteins is unknown.

There are three main reasons why the integration of residue representation into a deep neural network (DNN) is suitable for protein–protein interaction prediction. First, the principle of Word2vec technique considers the effect of a single word on surrounding words, which is consistent with the influence of a residue in the protein sequence on the surrounding residues. Previous studies have concluded that interaction residues exhibit aggregation to some extent (Ofran & Rost, 2003). It means that the probability for being an interacting residue will be high if it is close to an interaction residue. Therefore, Res2vec method can be used as an effective biological sequence representation tool. Second, representation learning can discover data-driven and abstractive features from raw data. It eliminates the noise of manual intervention. The represented vector can reflect comprehensive information in a protein sequence. Third, the arrangement of residues has an important effect on protein interaction prediction, it just so happen that DNN can learn the distribution of training data. In other words, DNN is able to capture high-level residue distribution features from the inputs. Above all, it is feasible to combine representation learning with deep learning to predict protein interactions.

## Materials and Methods

### Dataset

S.cerevisiae, Human and five species-specific protein–protein interaction datasets, which are collected from publicly available interacting protein databases (DIP) are used for performance evaluation. The S.cerevisiae core dataset (Guo et al., 2008) is chosen to confirm the parameters of the proposed model. The whole S.cerevisiae core dataset is made up of 11,188 protein pairs, including 5,594 positive protein pairs and 5,594 negative protein pairs. The human dataset (Huang et al., 2015) is also adopted to evaluate the effectiveness of the proposed method. It is collected from the Human Protein References Database (HPRD) and consists of 3,899 positive protein pairs and 4,262 negative protein pairs. To show the generality of model, five species-specific protein interaction datasets (C. elegans, E. coli, H. sapiens, M. musculus, and H. pylori) (Zhou, Gao & Zheng, 2011) are considered. These species-specific protein interaction datasets just contain positive samples. The positive interactions of each dataset are 4,013, 6,954, 1,412, 313 and 1,420.

### The framework of DeepFE-PPI method

**Figure 1:**
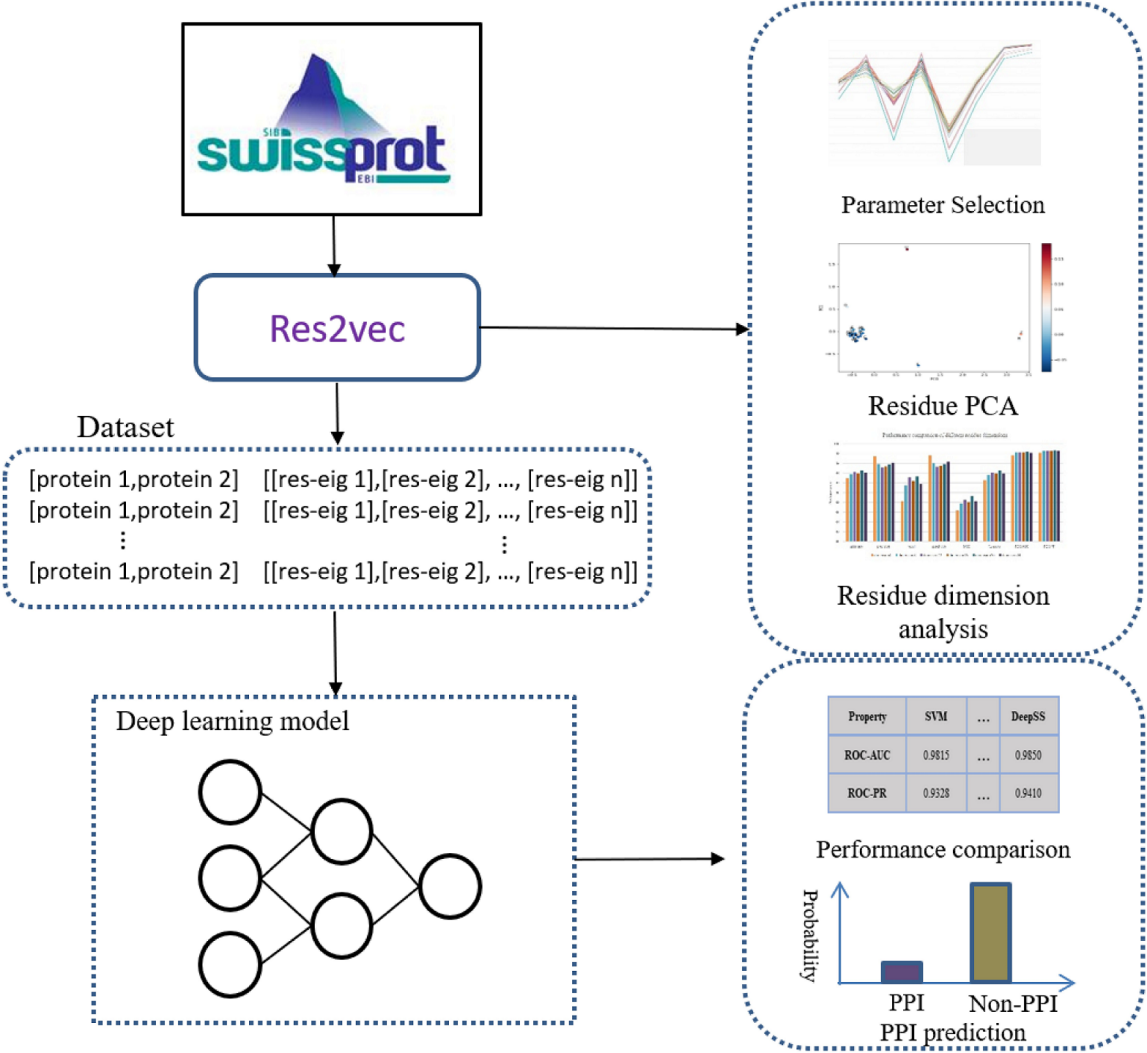
The general framework of DeepFE-PPI. In the represetation part, each type residue is represented by fixed-length vectors and proteins are uniformed to the same length. In the classification part, DNN is utilized to accomplish the classification task.

Representation learning can discover informative representations in a self-taught manner. Deep learning architecture can utilize multiple hierarchical layers to extract effective features. Leveraging the advantages of representation learning and deep learning, we proposed DeepFE-PPI framework, which consists of two main steps (Fig. 1): (1) residue representation learning; and (2) protein–protein interaction classification by deep learning.

### Residue representation learning

Word2vec has been a very successful technique for representation learning in an unsupervised way. Word representations can reflect item meaning using the co-occurrence information of words in a corpus. The fixed dimensionality representations generated by Word2vec have been shown to be very useful for various NLP task. Except for processing words in text processing field, Word2vec can map other information units (for example, phrase, sentences or documents) to a low-dimensional implicit space. In this implicit space, the representation of each information element is a dense eigenvector. Supplemental Information (Section: Continuous Skip-gram Model) gives the details of Word2vec.

This motivates us to transform each residue into a fixed dimensionality eigenvector representation. In previous work, many common word definition and splitting approaches in bioinformatics are proposed (Dong, Wang & Lin, 2005; Tsubaki, Shimbo & Matsumoto, 2017). Tsubaki, Shimbo & Matsumoto (2017) adopted Word2vec to learn feature representations from a large protein database to address protein fold recognition problem. However, previous work all define a word as an n-gram item, and they all split the sequences into overlapping of the n-gram residues. 20 types of amino acids make up a protein sequence, the total number of possible n-grams is 20^*n*^. The larger n, the larger vocabulary size in the corpus. More vocabularies require more run time and memory consumption when training the Word2vec model. So, we set n as 1, which means that a residue is treated as a word and a protein sequence is treated a sentence. Compared to other protein representation methods, (1) this method can obtain different residue interactions from the original protein corpus. (2) the cost of run time and memory consumption is acceptable.

In the training process of word representation, a large corpus of sentences should be fed into the model to ensure sufficient contexts are observed. In order to derive the representation of different biophysical and biochemical properties that hidden in protein sequences, 558,590 protein sequences from Swiss-Prot database are adopted as the input of the Word2vec model. The protein sequences were downloaded in Nov. 2018. There are two available models in Word2vec: the continuous bag of word (CBOW) model and the Skip-gram (SG) model. According to previous studies (Smaili, Gao & Hoehndorf, 2018), it is found that the SG model has advantage on creating better quality vector representations. Therefore, this paper uses the Skip-gram model to derive residue vectors.

**Figure 2:**
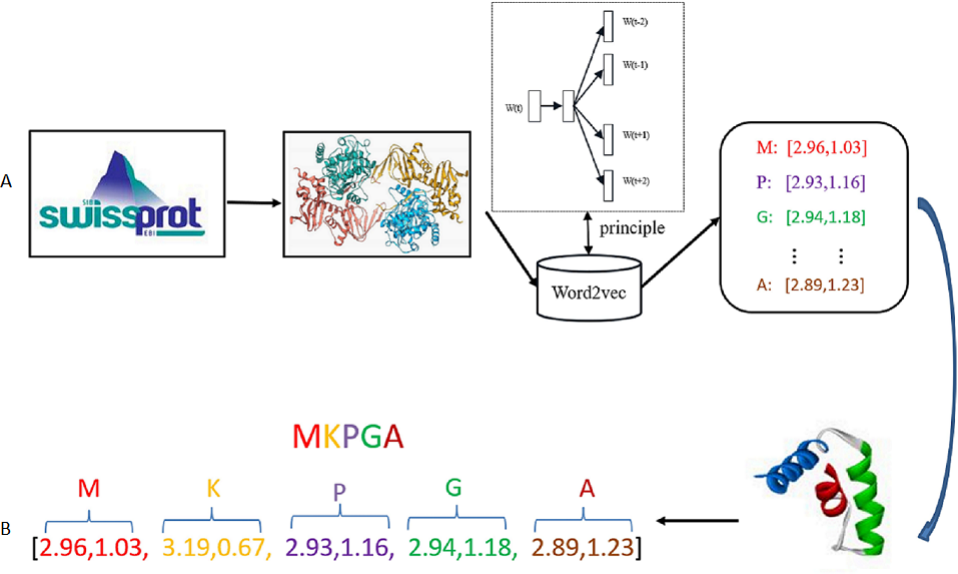
Residue representation learning. (A) The raw input data: each sample is a protein sequence. (B) Data transformation: each protein in the sample is represented as a concatenation of all residue vectors.

In this paper, Word2vec has been implemented using Gensim library. Hyper-parameters should be decided in the training stage. Hyper-parameters selection is a task dependent on choice and can vary from task to task. In the Skip-gram model, the target words are the words in the context, size denotes residue dimension, window denotes the maximum distance between the current and predicted word, min-count means the number of the ignored words with total frequency lower than a fixed value. Since the Swiss-Prot database just contains 25 different type residues, so the value of min-count in our experiment is set as 0. Parameters size and window are considered as the decisive parameters in the training of the Skip-gram model. After comparison of different parameter combinations, the value of size is set to be 20 and window is set as 4. The specific Word2vec parameters selection process is shown in Section Parameter selection of Results. Figure 2 shows the general process of residue representation. Protein sequences from Swiss-Prot database are adopted to train the Word2vec model. The output of the Word2vec model are residue eigenvectors. Each protein sequence can be represented as a concatenation eigenvector of residue vectors.

### Deep learning

Deep learning supports highly flexible architectures, such as DBNs (Deep Belief Networks), CNNs (Convolutional Neural Networks), RNNs (Recurrent Neural Networks) and so on. Different deep learning architectures are suitable for different specific applications. For example, DBNs are good at analyzing internal correlations in high-dimensional data, CNNs are capable of capturing complex features, and RNNs are suitable for the analysis of sequential information (Goodfellow, Bengio & Courville, 2016). Considering residues’ interactive impacts on each other and the possible high dimension of a protein sequence, we select DNN as the most appropriate or ‘best fit’ deep learning architecture to predict protein–protein interactions. Supplemental Information (Section: Deep neural network) gives the details of DNN.

**Figure 3:**
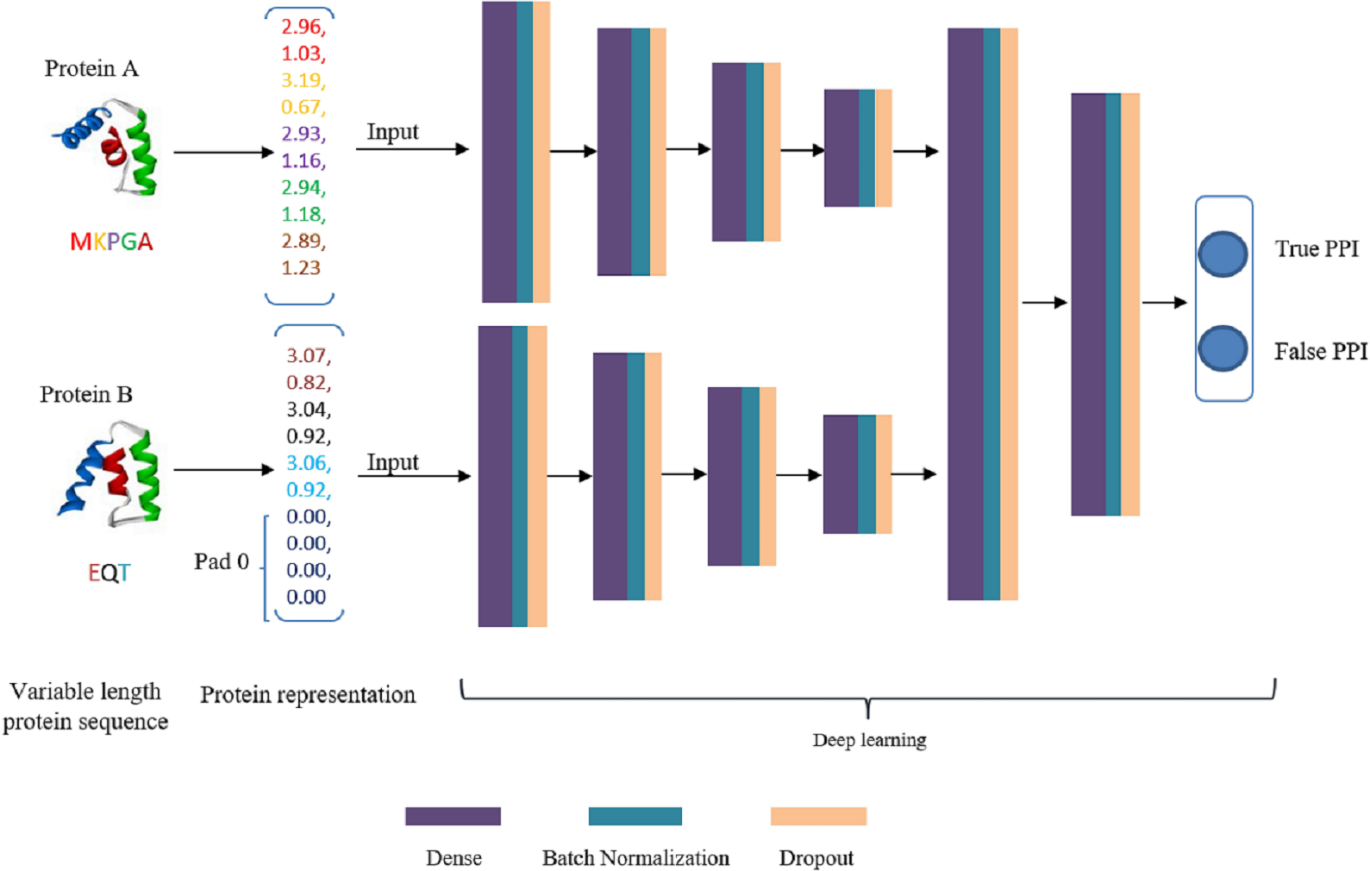
The framework of deep learning.

DNN is a very successful machine learning technique for classification in biology, but its input should be vectors with fixed dimension. This is a dreadful limitation because proteins are intrinsically expressed as sequences of arbitrary length. A simple measure is to uniform the length to a fixed size *m*. As a result, every protein sequence is represented as a vector of size *D*∗*m* (*D* is residue dimension and *m* is the fixed number of residues). If the length of a protein sequence is shorter than *m*, zero is appended to the short eigenvector to ensure every protein sequence has the same shape. These two embedding vectors are taken as input data of deep learning model. Our deep learning framework is composed of two separate DNN modules to extract high-level features that hidden in original embedding vectors and a joint module to integrate both DNN modules outputs to classify the input protein pair as interaction or non-interaction. The deep learning flowchart is shown in Fig. 3. For both DNN modules, we configure the same number of layers and hidden units. The number of fully connected layers is set as 4 and the corresponding units in each layer is 2,048, 2,014, 512 and 128, respectively. The main role of DNN module is to extract high-level feature, eliminate noise and reduce data dimension. The joint module takes the concatenated last hidden vectors of the both DNN modules as input via two fully connected hidden layers. The output layer utilizes a softmax function to predict interaction probability. In DeepFE-PPI, a batch-normalization layer and a dropout layer are added to each fully connected layer except the output layer. Rectified Linear Unit (ReLU) function is chosen for all layers except the final layer. During training, the models are optimized using Stochastic Gradient Descent (SGD) algorithm. L2-regularization term is added to the loss function as well, by adding the sum of the squares of all weights in the network.

Generally speaking, we first use unsupervised representation learning method Res2vec to generate residue representations. Then each protein sequence is uniformed into a vector representation with the same length. After that, separate DNN is established for feature extracting, the merged layer is for feature integration and the last layer is for classification.

## Results

In our study, all experiments are evaluated by cross validation and different metrics. For their introduction, please refer to the section (Cross validation and Performance evaluation) in the Supplemental Information.

### Parameter selection

We perform the protein–protein interaction prediction by the derived Res2vec model for residue vectors generation and deep learning technology for classification, therefore, there have two parameter sets needed to be confirmed. Previous literature (Li et al., 2018) has shown that training corpus size, residue vector dimension, and the algorithm adopted in Word2vec are the main factors that affect the quality of residue representations and the deep learning classification results. In addition, the hyperparameters of the deep learning model also determine prediction results. In order to get the optimal residue eigenvectors and deep learning configurations, we train the Skip-gram model on Swiss-Prot database which includes 558,590 proteins sequences and explore the effect on protein–protein interaction prediction over different parameter combinations on the S. Cerevisiae Core dataset. As the combinations of parameters increase exponentially and trying all of them is impossible in practice, we choose the most important hyper-parameters such as residue dimension, window size, network depth and protein length. Table 1 summarizes the most important hyper-parameters and their value intervals.

**Table 1:** Parameters used for determining the parameters of DeepFE-PPI and the final recommended settings.

**Parameters**	**Definition**	**Range**	**Optimized**
Sizes	Residue dimension	[4, 8, 12, 16, 20]	20
Windows	Maximum distance between the current and predicted word	[4, 8, 16, 32]	4
Min_count	Words with frequency lower than this value will be ignored	0	0
Maxlens	The length of proteins that feed to deep learning model	[550, 650, 750, 850]	850
Network depth	The hidden depth in the separate DNN model	[2, 3, 4, 5]	4

In the Skip-gram model, the two parameters of window size and residue dimension are most important. As the propensity for protein interactions is not a local property of single amino acid positions, it is expected that using the characteristics and patterns of neighboring residues could be helpful in the prediction of protein interactions. Previous studies (Murakami & Mizuguchi, 2010; Dhole et al., 2014) analyzed the entropy differences for various window lengths and finally emphasized that a nine-residue window size would be optimal for protein–protein interaction prediction problems, the initial length of the Skip-gram windows is taken as 4. Considering the run time and memory consumption, we train the residue vectors in case of window size [4, 8, 16, 32]. Residue dimension has a high impact on the classification, too. Because of our machine’s memory (16G) and residue dimension determines the size of a protein vector, residue dimensions are set as [4, 8, 12, 16, 20]. Other hyper parameters are set to default values in the toolkits of Word2vec. Table 2 shows the predictive performance of DeepFE-PPI on the S. Cerevisiae Core dataset with different combinations of residue dimension and window size. The experiment results show that when residue dimension is set as 20 and window size equals to 4, the model achieves the best performance with mean Accuracy 0.9461, mean Precision 0.9580, mean Recall 0.9333 and mean MCC 0.8927. The other measurements of Specificity, F1, AUC-ROC and AUC-PR are 0.9589, 0.9454, 0.9837 and 0.9868, respectively.

**Table 2:** Prediction performance of DeepFE-PPI on the S. Cerevisiae Core dataset with different combination of residue dimension and window size.

**Dimension-size**	**Accuracy**	**Precision**	**Recall**	**Specificity**	**MCC**	**F1**	**ROC-AUC**	**ROC-PR**
4–4	92.98%	97.45%	88.29%	97.68%	86.37%	92.63%	97.70%	98.21%
4–8	92.98%	97.27%	88.45%	97.52%	86.34%	92.64%	97.69%	98.21%
4–16	93.11%	97.26%	88.72%	97.50%	86.56%	92.79%	97.45%	98.06%
4–32	92.23%	96.74%	87.43%	97.03%	84.89%	91.82%	97.00%	97.71%
8–4	93.81%	95.89%	91.56%	96.05%	87.73%	93.66%	98.28%	98.58%
8–8	94.08%	96.23%	91.78%	96.39%	88.27%	93.94%	98.14%	98.52%
8–16	94.16%	96.58%	91.58%	96.75%	88.45%	94.01%	98.17%	98.54%
8–32	94.31%	95.25%	93.28%	95.33%	88.64%	94.25%	98.34%	98.67%
12–4	94.23%	95.21%	93.15%	95.30%	88.49%	94.16%	98.25%	98.59%
12–8	94.43%	96.76%	91.96%	96.91%	88.99%	94.29%	98.35%	98.66%
12–16	94.32%	96.27%	92.22%	96.42%	88.73%	94.20%	98.19%	98.53%
12–32	94.45%	96.89%	91.87%	97.03%	89.03%	94.30%	98.27%	98.62%
16–4	93.99%	95.41%	92.46%	95.53%	88.04%	93.90%	98.24%	98.56%
16–8	94.21%	95.37%	92.94%	95.48%	88.45%	94.14%	98.30%	98.64%
16–16	94.23%	95.98%	92.37%	96.10%	88.56%	94.13%	98.34%	98.63%
16–32	94.29%	95.70%	92.78%	95.80%	88.65%	94.20%	98.25%	98.59%
20–4	94.61%	95.80%	93.33%	95.89%	89.27%	94.54%	98.37%	98.68%
20–8	94.15%	94.94%	93.31%	94.99%	88.35%	94.10%	98.34%	98.64%
20–16	94.21%	95.40%	92.92%	95.50%	88.47%	94.13%	98.27%	98.57%
20–32	94.40%	96.52%	92.12%	96.67%	88.89%	94.26%	98.21%	98.54%

For deep learning, prediction results are determined by input data and model complexity. The deeper the DNN network is, the more abstract the features learned and the more accurate the model achieves. The selection of the network depth is related to the specific application and experimental data, so the appropriate network depth should be determined by experimental verification.

In the experiment, protein length is also an important factor that could influence the final result. We have experimented four different values of protein length starting from the average length with n step size as 100. The range of protein length is [n, n + 100, n + 200, n + 300]. So we discuss four different network structure depths in combination with different network inputs. The networks are incremented by one layer of fully connected layers. Tables 3, 4, 5 and 6 show that model performance increases first and then decreases when network depth increases from 2 to 5 with the condition that the protein length is set as 550, 650, 750 and 850. If network depth is not enough, the model is under-fitting and cannot capture deep features. While a network that is too deep will increase model complexity and training time. Due to protein length and the limitations of an experimental platform, DeepFE-PPI achieves the best performance when protein length is set as 850 and the number of layers in the network is 4.

**Table 3:** protein–protein interaction classification results under different depths with protein length 550.

**Depth**	**Accuracy**	**Precision**	**Recall**	**MCC**	**Specificity**	**F1**	**AUC- ROC**	**AUC- PR**
2	93.92	96.39	91.28	87.99	96.57	93.75	98.16	98.47
3	94.09	96.67	91.33	88.32	96.85	93.92	98.25	98.53
4	94.36	95.80	92.80	88.77	95.92	94.27	98.34	98.65
5	94.04	94.45	93.58	88.09	94.49	94.01	98.30	98.58

**Table 4:** protein–protein interaction classification results under different depths with protein length 650.

**Depth**	**Accuracy**	**Precision**	**Recall**	**MCC**	**Specificity**	**F1**	**AUC- ROC**	**AUC- PR**
2	93.92	95.96	91.71	87.93	96.14	93.78	98.05	98.36
3	94.40	96.76	91.88	88.93	96.93	94.26	98.23	98.53
4	94.34	95.06	93.56	88.71	95.12	94.30	98.33	98.65
5	94.02	94.64	93.33	88.05	94.71	93.98	98.26	98.60

**Table 5:** protein–protein interaction classification results under different depths with protein length 750.

**Depth**	**Accuracy**	**Precision**	**Recall**	**MCC**	**Specificity**	**F1**	**AUC- ROC**	**AUC- PR**
2	94.13	96.41	91.67	88.37	96.59	93.98	98.13	98.47
3	94.14	95.77	92.37	88.34	95.91	94.03	98.25	98.57
4	94.43	96.25	92.47	88.94	96.39	94.32	98.28	98.61
5	93.91	95.50	92.21	87.92	95.62	93.80	98.30	98.60

**Table 6:** protein–protein interaction classification results under different depths with protein length 850.

**Depth**	**Accuracy**	**Precision**	**Recall**	**MCC**	**Specificity**	**F1**	**AUC- ROC**	**AUC- PR**
2	93.88	95.97	91.62%	87.86	96.14	93.73	98.12	98.44
3	94.22	96.43	91.85	88.54	96.59	94.08	98.20	98.53
4	94.51	96.39	92.15	88.82	96.55	94.22	98.29	98.63
5	94.14	95.98	92.15	88.36	96.12	94.02	98.19	98.59

Hyper parameters are often selected in a statistically rigorous way via validation or cross-validation examples that are held out from training. We used five-fold cross-validation (training dataset proportion: 72%, validation proportion: 8%, testing proportion: 20%) and trained a neural network with four hidden layers on the S. Cerevisiae Core dataset. Usually, the training set is used to train models with different hyper-parameters, which are then assessed on the validation set. The best model configuration is then assessed by comparing the often used statistical measures, such as accuracy, recall, specificity, precision and MCC, on the test set.

### Performance comparisons with state-of-the-art algorithms

In order to evaluate the prediction performance of DeepFE-PPI, we compared the results of the proposed method with six state-of-the-art algorithms (Support Vector Machine (SVM), Random Forest (RF), Decision Trees (DT), Native Bayesian (NB), (K-Nearest Neighbor, KNN) and Logistic Regression (LR)). The experiments are carried on the S. Cerevisiae Core dataset using five-fold cross validation procedure. From methods in Li, Zhang & Liu (2017) and Maeda (2018), different machine learning models have very different algorithmic principles and parameters, only when comparing the algorithms with the most optimized parameters would make sense. Furthermore, training accuracy as well as training time should be compared as the evaluation criteria. For each algorithm, we fine-tune the model parameters by using grid-search to obtain the optimal parameters. Note, Naive Bayes algorithm model parameters via ‘partial-fit’ method and the sole parameter ‘priors’ uses default value that denotes the prior probabilities of the classes, so we have no training parameters for setting. Due to the fact that SVM performs slower than the other five algorithms, it takes about 20 h once time based on the configuration of our computer (a PC with an Intel Core i5-7400 processor and a 16G memory running windows 10), so the parameters are set to default values.The specific parameter details and the final recommended settings of each algorithm are described in Table 7.

**Table 7:** Different architecture with different types of parameters.

**Algorithm**	**Parameter**	**Range**	**Optimized**	**Accuracy**
KNN	n_neighbors	[2, 6, 10, 12, 16]	10	0.8336
DT	Criterion	[‘gini’,‘entropy’]	Gini	0.8218
LR	Penalty	[‘l1’,‘l2’]	L2	0.9261
RF	Estimators	[50, 150, 250, 350, 450, 550, 650, 750, 850, 950]	250	0.9293

**Figure 4:**
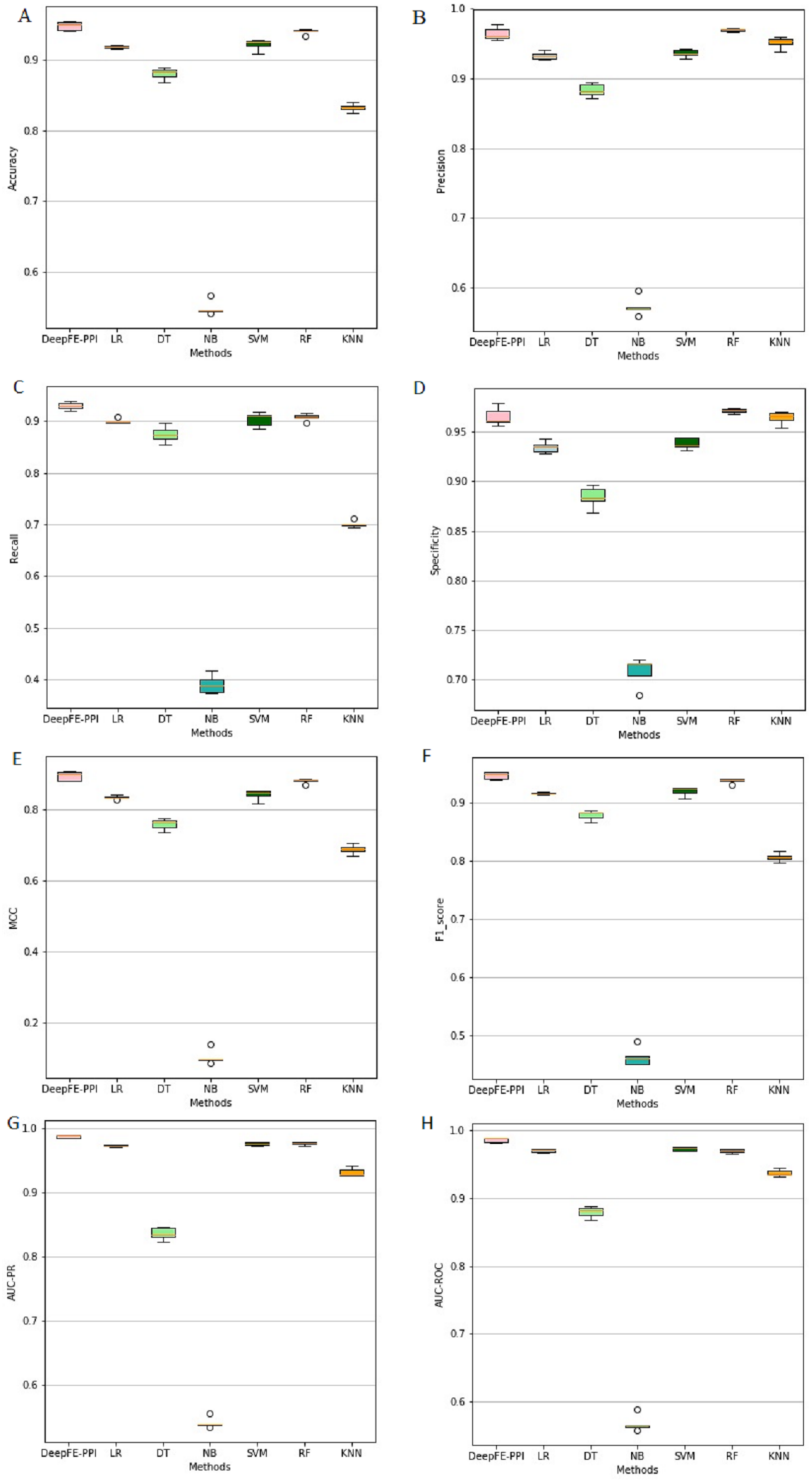
The classification results comparisons of DeepFE-PPI with state-of-the-art algorithms. (A) Accuracy on seven methods; (B) precision on seven methods; (C) recall on seven methods; (D) specificity on seven methods; (E) MCC on seven methods; (F) F1_score on seven methods; (G) AUC-PR on seven methods; (H) AUC-ROC on seven methods.

The proposed deep learning method and the other six different classification results are listed in Fig. 4. Among six state-of-the-art methods, Random Forest (RF) achieves 93.96% accuracy, 96.96% precision, 90.76% recall, 97.16% specificity, 88.10% MCC, 93.76% F1, 96.93% AUC-ROC and 97.69% AUC-PR. The classification results of DeepFE-PPI are 0.82% accuracy, −0.51% precision, 2.23% recall, −0.59% specificity, 1.52% MCC, 0.93% F1, 1.61% AUC-ROC and 1.14% AUC-PR higher than RF. Experimental results show that DeepFE-PPI performs better in detecting protein–protein interactions in terms of accuracy, recall, MCC, F1, AUC-ROC and AUC-PR.

From Fig. 4, we can see that the result of RF is also excellent. However, this method requires manual selection of features. Firstly, it takes a lot of time to find suitable features. If the features are not found properly, the results will be affected. Second, feature selection and data training are a process of separation and cannot assist each other. The main advantage of the deep learning is the automation of feature selection. It can automatically process the features that can express the essence of things according to the follow-up training process, so as to realize the automation process without manual intervention. Furthermore, RF is sensitive to the noise in the original input, frequent noise brought by features construction from the low-level data may make the subsequent classifier to learn wrong knowledge. While a deep learning framework consists of neural networks stacked together (Hinton & Salakhutdinov, 2006), the stacked layers help to reduce the noise effects in the original input. Due to limitations in our computer hardware configuration, the deep learning model we constructed is not strong enough, we believe that deep learning model can perform better than RF in all indexes if the hardware configuration is better.

Table 8 shows training time with the most optimized parameters between different algorithms. As seen from Table 8, the time of DeepFe-PPI ranks fourth in all seven methods. Naive Bayes training time is the shortest, but its results are the worst.

### Performance comparisons with existing methods on the S. Cerevisiae Core Subset

Our analysis first proceeds on the S. Cerevisiae Core dataset. The results of different methods with five-fold cross validation on the S. Cerevisiae Core dataset are shown in Table 9. From Table 9, we can see that DeepFE-PPI achieves good prediction performance with a 94.78% average accuracy, 92.99% average recall, 96.45% average precision and 89.62% average MCC. Compared with our previous proposed deep learning-based algorithm DeepPPI, DeepFE-PPI boosts about 0.35% accuracy, 0.93% recall, −0.20% precision and 0.65% MCC. However, DeepPPI used five different category features to represent a sequence of a protein. The feature extraction process is more complicated than Res2vec, which directly represent a residue. Both on classification results and protein representation step, DeepFE-PPI performs better than Deep-PPI. From the overall perspective, DeepFE-PPI outperforms better than most other approaches (10 of the 13 methods with the whole measure metrics). But it seems that (Wang et al., 2017b) obtained the highest performance according to each evaluation metric and the following one is RFEC (Song et al., 2018). Note that, the Legendre moments (LMs) (Wang et al., 2017b), Zernike moments (ZM) descriptor (Wang et al., 2017a) and the evolutionary information (Song et al., 2018) all extracted discriminatory information embedded in the position-specific scoring matrix (PSSM) which is generated by Position-Specific Iterated Basic Local Alignment Search Tool (PSI-BLAST) (Altschul et al., 1997). Above three methods run much slower because it is required to run BLAST against a huge protein NR database to generate a PSSM matrix as its feature. For Huang et al. (2015), the performance on the S. Cerevisiae Core dataset is really better than DeepFE-PPI while the performance on the human and five independent datasets are worse than DeepFE-PPI. Especially that using the S. Cerevisiae Core dataset as training set and five independent across species datasets as test sets, the accuracies of the approach Huang et al. (2015) proposed are 33.92% (E.coli), 18.81% (C. elegans), 17.78% (H. sapiens), 17.82% (H. pylori), and 20.13% (M.musculus) lower than DeepFE-PPI, which indicates that the generalizability of Huang’s model (Huang et al., 2015) is quite weak. More details of independent datasets are shown in Table 9. Considering computational time, feature extraction process and model generalizability, DeepFE-PPI is more appropriate for protein–protein interaction prediction.

**Table 8:** The time with the most optimized parameters between different algorithms.

**Classifier**	**SVM**	**DT**	**RF**	**NB**	**LR**	**KNN**	**DeepFE-PPI**
Time (second)	>92365.99	890.72	696.06	48.52	2292.30	6593.53	1008.16

**Table 9:** Performance comparisons of 14 methods on the S. Cerevisiae Core dataset.

**Method**	**Feature**	**Classifier**	**Accuracy**	**Recall**	**Precision**	**MCC**
DeepFE-PPI	Res2vec	DL	94.78 ± 0.61	92.99 ± 0.66	96.45 ± 0.87	89.62 ± 1.23
Huang et al. (2015)	DTC+SMR	WSRC	96.28 ± 0.52	92.64 ± 1.00	99.92 ± 0.18	92.82 ± 0.97
Wang et al. (2017b)	PSSM	PCVM	96.37 ± 0.20	96.60 ± 0.60	96.15 ± 0.50	93.00 ± 0.40
Wang et al. (2017a)	PSSM	PCVM	94.48 ± 1.20	95.13 ± 2.00	93.92 ± 2.40	89.58 ± 2.20
Song et al. (2018)	PSSM	RFEC	95.64 ± 0.52	94.47 ± 0.47	96.75 ± 0.45	91.30 ± 1.03
You, Chan & Hu (2015)	MLD	RF	94.72 ± 0.43	94.34 ± 0.49	98.91 ± 0.33	85.99 ± 0.89
Du et al. (2017)	Mutiple	DL	94.43 ± 0.30	92.06 ± 0.36	96.65 ± 0.59	88.97 ± 0.62
You et al., 2013)	Mutiple	PCA-EELM	87.00 ± 0.29	86.15 ± 0.43	87.59 ± 0.32	77.36 ± 0.44
You et al. (2014)	MCD	SVM	91.36 ± 0.36	90.67 ± 0.69	91.94 ± 0.62	84.21 ± 0.59
Wong et al. (2015)	PR-LPQ	RoF	93.92 ± 0.36	91.10 ± 0.31	96.45 ± 0.45	88.56 ± 0.63
Guo et al. (2008)	ACC	SVM	89.33 ± 2.67	89.93 ± 3.68	88.87 ± 6.16	N/A
Guo et al. (2008)	AC	SVM	87.36 ± 1.38	87.30 ± 4.68	87.82 ± 4.33	N/A
Zhou, Gao & Zheng (2011)	LD	SVM	88.56 ± 0.33	87.37 ± 0.22	89.50 ± 0.60	77.15 ± 0.68
Yang, Xia & Gui (2010)	LD	KNN	86.15 ± 1.17	81.03 ± 1.74	90.24 ± 1.34	N/A

### Performance comparisons with existing methods on the human dataset

Consequently, human dataset is employed as a test dataset. The average prediction results with five-fold cross-validation over twelve different approaches are given in Table 10. Table 10 clearly shows that DeepFE-PPI achieves the highest performance in terms of accuracy (98.71%), recall (98.54%) and MCC (97.43%). Compared to our previous work (Du et al., 2017), the value of accuracy, recall and MCC has improved 0.57%, 1.59% and 1.14%, respectively. The second-best value (98.77%) of precision is just slightly lower (−0.36%) than our previous work (99.13%). Since our previous work has achieved best in predicting protein–protein interactions, so taken together, this comparative analysis demonstrates that residue representations detected by Res2vec achieve comparable classification performance, in contrast to sophisticated feature extraction step.

**Table 10:** Performance comparisons of 12 methods on the human dataset.

**Methods**	**Feature**	**Classifier**	**Accuracy**	**Recall**	**Precision**	**MCC**
DeepFE-PPI	Res2vec	DL	98.71 ± 0.30	98.54 ± 0.55	98.77 ± 0.53	97.43 ± 0.61
Ding, Tang & Guo (2016)	MMI+NMBAC	RF	97.56	96.57	98.30	95.13
Ding, Tang & Guo (2016)	MM1	RF	96.08	95.05	96.97	92.17
Ding, Tang & Guo (2016)	NMBAC	RF	95.59	94.06	96.94	91.21
Du et al. (2017)	Mutiple	DL	98.14	96.95	99.13	96.29
Huang et al. (2015)	DTC + SMR	WSRC	96.30	92.63	99.59	92.82
Liu et al. (2015)	LDA	RF	96.4	94.2	N/A	92.8
Liu et al. (2015)	LDA	RoF	95.7	97.6	N/A	91.8
Liu et al. (2015)	LDA	SVM	90.7	89.7	N/A	81.3
Liu et al. (2015)	AC	RF	95.5	94.0	N/A	91.4
Liu et al. (2015)	AC	RoF	95.1	93.3	N/A	91.0
Liu et al. (2015)	AC	SVM	89.3	94.0	N/A	79.2

### Performance comparisons on independent datasets

To further validate the performance of our proposed method, we also compare the predictive performance of our method on five other independent species datasets. In this experiment, the selected 11,188 samples of S. cerevisiae core subset are used as training data, five other species datasets (E. coli, C. elegans, H. sapiens,H. pylori and M.musculus) are used to assess the performance of DeepFE-PPI. Table 10 gives the detailed performance comparisons among different methods. The value of accuracies are 100.00% (E. coli), 100.00% (C. elegans), 100.00% (H. sapiens), 100.00% (H. pylori), and 100.00% (M.musculus) by DeepFE-PPI, respectively. From Table 11, we can see that DeepFE-PPI is superior to DeepPPI on all five across species independent datasets. Compared with the other methods, our method obtains the highest accuracy on five independent species datasets. The promising results demonstrate that the proposed method has higher accuracy and better generalizability.

**Table 11:** Performance comparisons (Accuracy) on five independent datasets.

Method	E.coli	C. elegans	H. sapiens	H. pylori	M.musculus
**DeepFE-PPI**	**100**	**100**	**100**	**100**	**100**
Du et al. (2017)	92.19	94.84	93.77	93.66	91.37
Huang et al. (2015)	66.08	81.19	82.22	82.18	79.87
Zhou, Gao & Zheng (2011)	71.24	75.73	76.27	N/A	76.68
Wang et al. (2017b)	92.80	92.60	80.10	N/A	89.14
Ding, Tang & Guo (2016)	92.80	92.16	94.33	91.13	95.85
Ding, Tang & Guo (2016)	89.01	88.54	91.31	90.28	92.01
Ding, Tang & Guo (2016)	90.13	86.72	90.23	90.34	91.37
You, Chan & Hu (2015)	89.30	87.71	94.19	90.99	91.96

### Park and Marcotte’s evaluation scheme

Typical cross-validation for pair-input methods divide available data into a training set and a test set, ignoring that the way of test datasets constructed can significantly impact the performance of the pair-input predicting programs (Park & Marcotte, 2012). Based on the component-level overlap of test datasets, test dataset can be partitioned into three distinct classes: C1, test pairs sharing both proteins with the training set; C2, test pairs sharing only one protein with the training set; C3, test pairs sharing neither protein with the training set. We design similar testing datasets according to Park and Marcotte’s procedure (Park & Marcotte, 2012). S. Cerevisiae Core dataset has 2,530 different protein sequences. We randomly split the whole protein sequences into two parts: part A has protein sequences (numbers) while the rest serve as part B. Then we pick out two interacting protein sequences sharing both proteins in the 2,230 protein sequence as training and C1 type datasets (4/5 as training dataset and 1/5 as C1 type dataset). Protein pairs that one protein sequence in part A and the interacting protein sequence in part B is considered as C2 type dataset. Protein pairs that both from part B constitute C3 type dataset. The performance on each dataset is showed as follows (Table 12):

**Table 12:** The experiment results on C1, C2 and C3 type.

**Partition**	**Accuracy**	**Precision**	**Recall**	**Specificity**	**MCC**	**F1**	**AUC- ROC**	**AUC- PR**
C1	94.48	96.95	91.69	97.19	89.07	94.24	98.24	98.59
C2	78.50	78.01	84.92	70.62	56.39	81.32	88.03	90.13
C3	72.73	77.55	86.36	40.00	29.29	81.72	71.28	85.50

### Visualization of residue representations

**Figure 5:**
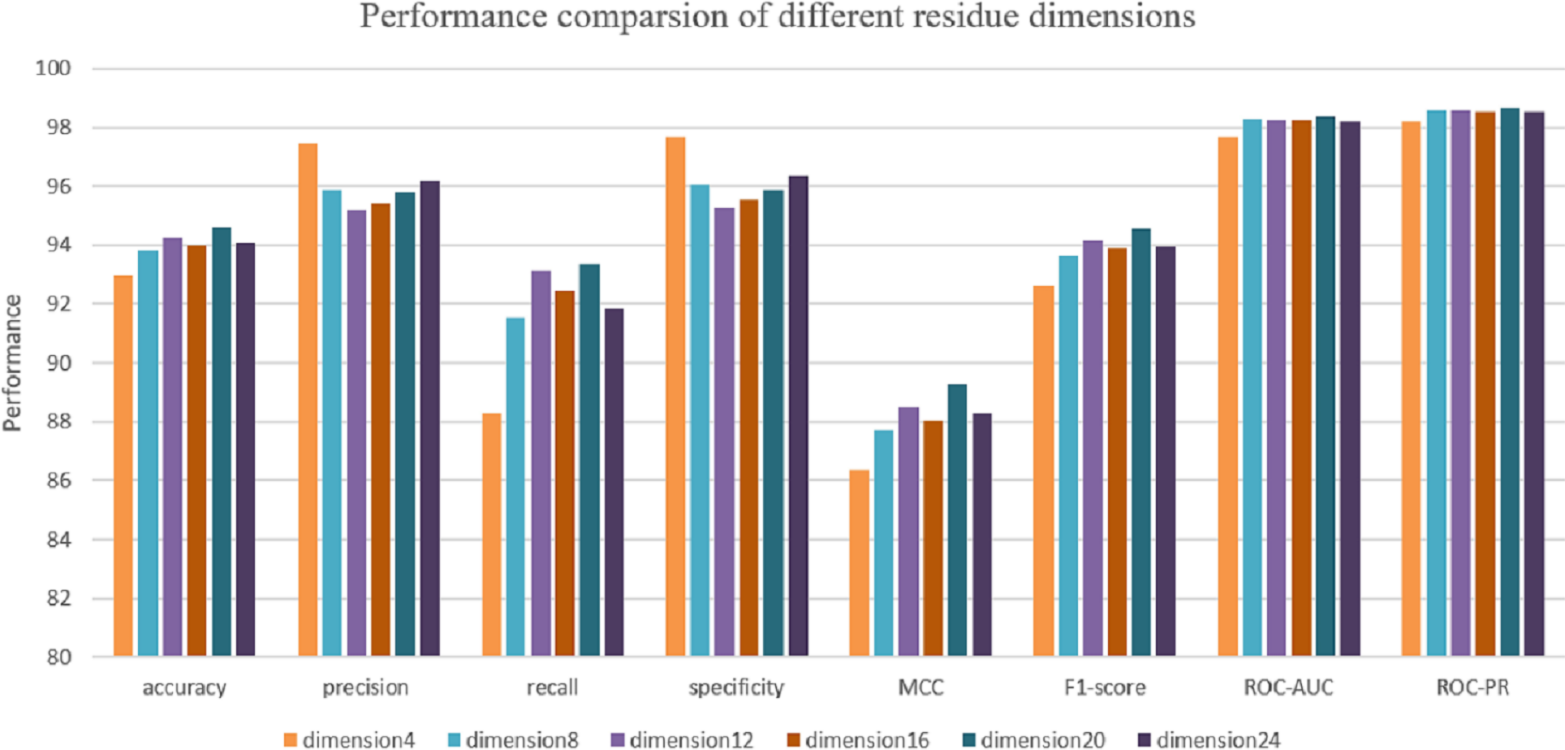
The results corresponding to residue with dimension 4, 8, 12, 16, 20, 24.

Due to the input sequence representation determines the regulatory properties of each protein. Hence, it is critical to determine the optimal dimension of each residue for protein–protein interaction prediction. Maintaining a consistent data split ratio with five-fold cross validation, 72% of the dataset is split as a training set, 8% of the dataset is split as a validation set and 20% for model testing. We use DeepFE-PPI model to predict interactions on the S. Cerevisiae Core dataset and obtain 6 performance results under 6 residue eigenvector sets. Each result corresponds to a special residue dimension (from 4 to 24 with 4 steps). The performance with different residue dimensions is shown in Fig. 5. From Fig. 5, we can see that when the residue’s dimension increases from 4 to 20, the whole performance measure metrics (accuracy, recall, precision and MCC) is gradually rising. The classification results decrease when the residue dimension continues to increase four steps starting from 20. DeepFE-PPI achieves superior performance with the dimension of residue equals to 20. Considering run time and memory size, the optimal residue dimension in this article is selected as 20.

**Figure 6:**
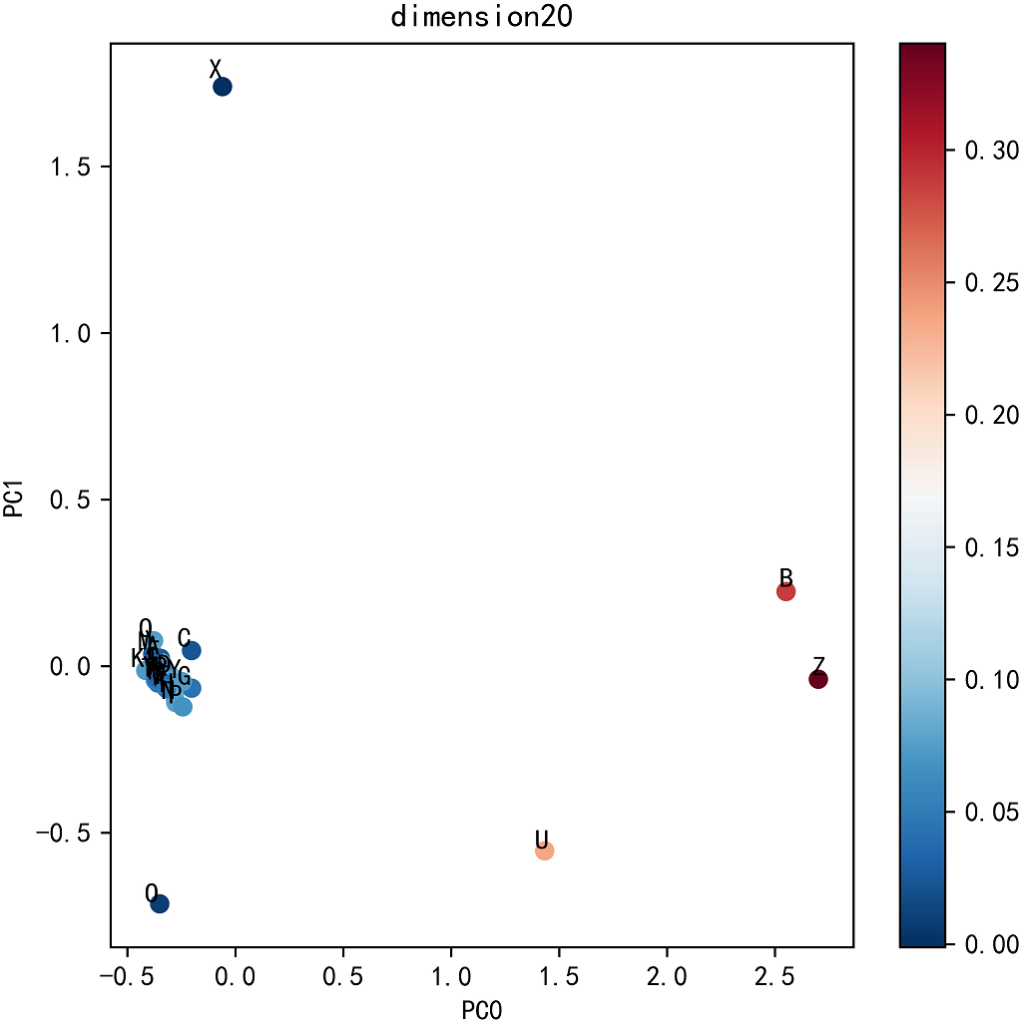
Residue PCA.

To visualize the effect of residue dimension for residue quality, we applied principal component analysis on the optimal dimension residues. The top five ratios of the variance of each PCs are 0.6421, 0.1412, 0.0649, 0.0347, 0.0279. The first two principal components are adopted to cluster 25 residues. As can be seen from Fig. 6, the common 20 residues are clustered together while the other five uncommon residues are far apart, which also explains the rationality of Res2vec.In addition, we also show the memory and run time (please see Supplemental Information: Memory and Time).

## Discussion

Protein–protein interaction prediction has been studied for many years. The main techniques used in this study is Res2vec for feature representation and deep learning for classification. The prediction results on different datasets indicate that Res2vec does as well or better than other complex feature extraction approaches and DeepFE-PPI has a certain help in protein–protein interaction prediction problem.

The key to DeepFE-PPI framework is the use of Word2vec, which is capable of generating continuously sequence representation. Different residue representations generated by Res2vec might induce the following deep learning network to produce particularly poor or good results. Choosing an appropriate residue dimension is the key but we have not found an effective chosen method yet. In the parameter analysis section, we try to use principal component analysis to find the relationships between residues. Unfortunately, we only find that the common 20 residues cluster together while the other five uncommon residues are far away. From this phenomenon, we then choose the optimal residue dimension by evaluating the performance of the classifier as a criterion, not from the feature generator Word2vec. Based on this, there is a lot of work that can be done about how to choose the optimal residue dimension and why the selected dimension can induce the best classification for protein interaction prediction problem.

## Conclusions

Protein–protein interaction prediction is important for understanding the activity of complex cells from a molecular point of view. In this work, we propose a new protein sequence representation method combined with an effective deep learning framework to predict protein–protein interactions. Inspired by word embedding in natural language processing and leveraging large scale positive protein data, the proposed residue representation method Res2vec captures a diverse range of meaningful properties and represent each type residue as a low-dimensional expressive vector from raw data solely on protein sequence information. Then two individual DBN modules are used to extract high-level feature from each protein and a joint module to identify whether two proteins interact with each other or not. The results show that the combination of effective residue representation strategy and powerful deep learning technique is particularly useful for protein–protein interaction prediction. Comparisons with other published classifiers show that DeepFE-PPI is capable of predicting protein–protein interactions by a novel residue representation method and an effective deep learning framework in an acceptable level of accuracy.

##  Supplemental Information

10.7717/peerj.7126/supp-1 Supplemental Information 1 Supplemental Materials Continuous Skip-gram Model, Deep neural network, Cross validation, Performance evaluation, Memory and Time.

## Additional Information and Declarations

### Data Availability

The following information was supplied regarding data availability: Data is available at GitHub: https://github.com/xal2019/DeepFE-PPI.
