# Supplementary material for: An integration of deep learning with feature embedding for protein–protein interaction prediction
Source: PeerJ. 2019 Jun 17;7:e7126. doi: 10.7717/peerj.7126 (PMC6585896; doi:10.7717/peerj.7126)
Supplement: Supplemental Information 1 — Continuous Skip-gram Model, Deep neural network, Cross validation, Performance evaluation, Memory and Time. [file peerj-07-7126-s001.pdf]

SUPPLEMENTAL MATERIALS

Continuous Skip-gram Model

Word2vec is a successful word embedding technique in various applications in NLP. The main idea of Word2vec is to convert a word into a N-dimensional continuous vector. It can store information of an item within a system through establishing interactions with other members, i.e. neighboring words. Word2vec implements two main architectures, continuous bag-of-words (CBOW) model and the continuous Skip-gram (SG) model, to derive vector representations for different type words in the corpus. The input layer of CBOW model is the context words and the goal is to maximize the probability of the centered word based on the context words, while the Skip-gram model takes the current word as input and the output layer maximizes the likelihood of words within a certain range before and after the current word (Mikolov et al., 2013).

In this work, the continuous Skip-gram model is adopted as the training model. In the Skip-gram model, the words occurring further away from the current word are given less weight while the close ones are endowed higher weight. The objective function of the Skip-gram model is shown in equation (1) and the conditional probability function is shown in equation (2):

$$L = \sum_{w \in C} \log p(Context(w)|w) \tag{1}$$

$$p(Context(w)|w) = \prod_{u \in Context(w)} p(u|w) \tag{2}$$

where C denotes all the words in a context window, w is the centered word in the window, $Contexw(w)$ denotes the words in the context window except the centered one, and Equation (1) is viewed as the log-likelihood of a given sentence set.

From the performance of the two models, the CBOW model takes a shorter time for training and the accuracy is slightly lower. Word vectors produced by the Skip-gram model have higher accuracy and the Skip-gram model has advantages to deal with low-frequency words.

Deep neural network

A deep neural network (DNN) can be viewed as a mathematical function that receives data in an input layer, then transforms the inputs in a nonlinear way through multiple sequential layers, and offers outputs in the final layer (Wainberg et al., 2018; Angermueller et al., 2016). For a standard two-layer neural network, given an input vector v=[v_i], we can calculate the predictive value y_k from a mathematical function as follows:

$$y_k(v;\theta) = f^{(2)}(\sum_{j=1}^{m} W_{kj}^{(2)} f^{(1)}(\sum_{i=1}^{D} W_{ji}^{(1)} v_i + b_j^{(1)}) + b_k^{(2)}) \tag{3}$$

where v_i is the i-th element of the input vector, m is the number within hidden units,W_{ij}^{n} denotes the weight of the i-th neuron of the n-th layer and the j-th neuron of the adjacent layer, b^n represents the bias of the n-th layer. θ=$\{W^{(1)}, W^{(2)}, b^{(1)}, b^{(2)}\}$ denotes model parameters, these parameters can capture the distribution from input/output samples. $f^{(1)}(\cdot)$ and $f^{(2)}(\cdot)$ denote nonlinear activation functions that can guarantee the nonlinearity of the whole model.

The goal of model training is to derive the most appropriate parameters θ so that when a new sample is given, the probability measured at the output is heavily skewed to the correct class. A single cycle of the parameter learning process is organized as (LeCun et al., 2015). Given a training dataset, the forward pass sequentially computes the output in each layer and propagates the function signals forward through the network. In the final output layer, an objective loss function measures error between the predicted outputs and the true labels based on the current set of model weights. The loss is then backward propagated through the chain rule to compute the gradients of the loss function. The model updates the parameters iteratively. Backpropagation (Werbos, 1990) determines how much to adjust each weight and the gradient can be efficiently evaluated by the object loss function. The formula of parameters update process is shown as follows:

$$\theta^{\tau+1} = \theta^{\tau} - \eta \bigtriangledown E(\theta^{\tau}) \tag{4}$$

where $\bigtriangledown E(\cdot)$ is a gradient vector of all the layers, η is a learning rate and τ denotes an iteration index. The update process is repeated until convergence. The weight parameters are often updated using stochastic gradient descent (SGD) algorithm with a minibatch subset of training samples (Bottou, 1991).

In the canonical configuration, the object loss function of a classification network is often a cross entropy loss function:

$$H(p,q) = -\sum_{x} p(x) log\ q(x) \tag{5}$$

It defines between a true distribution and the estimated class probabilities.The most popular hidden units’ activation function is the Rectified Linear Unit (ReLU) function. This type of activation function thresholds negative signals to 0 and passes through positive signal. Such operation allows faster learning compared to other alternatives functions.

$$ReLU(x) = max(0,x) \tag{6}$$

The output units’activation function is dependent on the target task being a classification or regression problem. For example, a softmax activation function works as the following equation:

$$f_i(z) = \frac{e^{z_i}}{\sum_j e^{z_j}} \tag{7}$$

The value of f_i(z) denotes the predicted score for class i.

In addition, regularization plays an important role in the training of deep learning architectures. It can avoid over-fitting and thus achieve good generalization performance. Currently, the most widely used regularization approach is dropout (Srivastava et al., 2014). Dropout operation removes hidden units from neural networks randomly during the training stage and can improve classification accuracy. Furthermore, batch normalization (Ioffe and Szegedy, 2015) provides a new regularization method through normalization of scalar features for each activation within a mini-batch.

Cross validation

Cross-validation scheme is a standard experimental technique for avoiding any sampling biases and verifying the constancy of the model (Stone, 1974). In this work, a five-fold cross validation procedure is applied for evaluating the performance of the classifier. More specifically, in a five-fold cross-validation procedure, the entire dataset is split into 5 random equal parts (folds), four of five folds are used for

training and the remaining one is used as test set in each fold of the cross validation. In this way, the same process is repeated five times and five separate models are obtained. Finally, the results on five separate experiments are averaged to give a comprehensive evaluation.

Performance evaluation

To assess the effectiveness and reliability of a classifier quantitatively, several widely used statistical measures, including accuracy, recall, specificity, precision, MCC (Matthew's correlation coefficient) and F1 are taken to perform performance evaluation. The precise definition of these measures is as follows:

$$Accuracy = \frac{TP+TN}{TP+TN+FP+FN} \tag{8}$$

$$Recall = \frac{TP}{FN+TP} \tag{9}$$

$$Specificity = \frac{TN}{TN+FP} \tag{10}$$

$$Precision = \frac{TP}{TP+FP} \tag{11}$$

$$MCC = \frac{TP\times TN - FP\times FN}{\sqrt{(TP+FP)(TP+FN)(TN+FP)(TN+FN)}} \tag{12}$$

$$F1 = \frac{2TP}{2TP+FP+FN} \tag{13}$$

where, TP represents true positives that are correctly predicted interacting proteins, FP represents false positives that are incorrectly predicted non-interacting proteins, TN denotes true negatives that are correctly predicted non-interacting proteins, FN means false negatives that are incorrectly predicted interacting proteins, respectively.

Among the above indexes, accuracy is a simple evaluation standard but there is a limitation that it would provide a very biased evaluation if the dataset is imbalance. Precision measure shows the number of predicted parts that are actually related to the protein-protein interactions. Since precision and recall are working in opposite direction, F1 is taken as the weighted harmonic mean of precision and recall to reflect the overall prediction performance (Hripcsak and Rothschild, 2005). A high value of F1 means a high value of both precision and sensitivity. MCC is an another objective index to reflect the overall performance of a method and can measure the matching degree between the prediction results and the real results (Matthews, 1975). In addition, the average AUC-ROC and AUC-PR value are also utilized to assess the performance of the classifier (Baten et al., 2006). AUC-ROC and AUC-PR compare the classifiers' performance across the entire range of class distributions and error costs.

Memory and Time

Table 1 shows the memory and run time. From Table 1, we can see that the memory space increases exponentially when the residue dimension increases, for computational sake, we set upper limit as 24 for residue dimension. In our study, the window size of the SG model is set to 4, and residue dimension is set as 20. It is noteworthy that, Res2vec gains superiority of the running time to represent residue vector in data representing. Res2vec dramatically saves much time and manpower. With the use of NVIDIA GeForce GTX 1060 3GB, each training epoch takes only about 376s. It just needs less than 2 hours to train the DeepFE-PPI framework. With the aid of computer hardware, DeepFE-PPI can allow researchers to derive a novel residue representation method and a deep learning-based classifier in a tolerable level short time.

Table 1. Runtime and memory consumption of Res2vec models with different parameters.

Parameters (size,window)	Word2vec Time (second)	Memory consumption
(4,4)	251.03	40.1% (6534M/16294M)
(4,8)	397.17	43.6% (7104M/16294M)
(4,16)	716.31	50.5% (8227M/16294M)
(4,32)	1147.23	55.6% (9053M/16294M)
(8,4)	246.89	63.5% (10340M/16294M)
(8,8)	352.94	71.4% (11638M/16294M)
(8,16)	598.37	78.0% (12711M/16294M)
(8,32)	1181.66	49.8% (8108M/16294M)
(12,4)	287.56	84.2% (13721M/16294M)
(12,8)	402.41	77.5% (12633M/16294M)
(12,16)	728.63	86.9% (14161M/16294M)
(12,32)	1193.26	72.2% (11770M/16294M)
(16,4)	228.15	34.3% (5592M/16294M)
(16,8)	433.25	53.4% (8700M/16294M)
(16,16)	729.31	59.9% (9755M/16294M)
(16,32)	1054.91	54.6% (8900M/16294M)
(20,4)	260.53	55.5% (9039M/16294M)
(20,8)	420.19	53.9% (8783M/16294M)
(20,16)	743.30	55.9% (9108M/16294M)
(20,32)	1258.02	53.0% (8642M/16294M)
(24,4)	242.17	44.0%(7161M/16294M)
(24,8)	381.04	58.5% (9525M/16294M)
(24,16)	754.39	64.2% (10466M/16294M)
(24,32)	1281.08	56.7% (9240M/16294M)

REFERENCES

Angermueller, C., P?rnamaa, T., Parts, L., and Stegle, O. (2016). Deep learning for computational biology. *Molecular systems biology*, 12(7):878.

Baten, A. K., Chang, B. C., Halgamuge, S. K., and Li, J. (2006). Splice site identification using probabilistic parameters and svm classification. *BMC bioinformatics*, 7(5):S15.

Bottou, L. (1991). Stochastic gradient learning in neural networks. *Proceedings of Neuro-N?mes*, 91(8):12.

Hripcsak, G. and Rothschild, A. S. (2005). Agreement, the f-measure, and reliability in information retrieval. *J Am Med Inform Assoc*, 12(3):296–298.

Ioffe, S. and Szegedy, C. (2015). Batch normalization: Accelerating deep network training by reducing internal covariate shift. *arXiv:1502.03167*, pages 448–456.

LeCun, Y., Bengio, Y., and Hinton, G. (2015). Deep learning. *Nature*, 521(7553):436–444.

Matthews, B. W. (1975). Comparison of the predicted and observed secondary structure of t4 phage lysozyme. *Biochim Biophys Acta*, 405(2):442–451.

Mikolov, T., Yih, W. T., and Zweig, G. (2013). Linguistic regularities in continuous space word representations. *Proceedings of the Conference of the North American Chapter of the Association for Computational Linguistics: Human Language Technologies*, pages 746–751.

Srivastava, N., Hinton, G., Krizhevsky, A., Sutskever, I., and Salakhutdinov, R. (2014). Dropout: a simple way to prevent neural networks from overfitting. *Journal of Machine Learning Research*, 15(1):1929–1958.

Stone, M. (1974). Cross-validatory choice and assessment of statistical predictions. *Journal of the royal statistical society. Series B (Methodological)*, 36(2):111–147.

Wainberg, M., Merico, D., Delong, A., and Frey, B. J. (2018). Deep learning in biomedicine. *Nature Biotechnology*, 36(9):829–838.

Werbos, P. J. (1990). Backpropagation through time: what it does and how to do it. *Proceedings of the IEEE*, 78(10):1550–1560.
